# Neuralgia

**Published:** 1871-12

**Authors:** Alexander Wilder


### ARTICLE VII.

### *Neuralgia.*

BY ALEXANDER WILDER, M. D.

From an Essay read before the Eclectic Medical Society of the city of New York.

Foremost among the ailments that afflict human beings in civilized countries is the pest formerly known as *tic douleureux*, but now denominated neuralgia. It has been known ever since Hippocrates. The complaint generally affects the first, second, and third branches of the fifth pair of nerves, and the *portio duro*, the left side of the face being oftener attacked, and the female sex more commonly afflicted. But persons of each sex will recognize it as a "mutual friend," and all parts of the body are subject to its invasion. We may have cerebral, cardiac, spinal, and ganglionic neuralgia. It is the defective nutrition, however occasioned, and an offspring of scrofula. We apprehend that it pertains almost exclusively to that diathesis. It is produced by miasmatic atmospheric changes, ozena, or catarrh of the head, bad teeth, imperfect digestion, mental excitement and anxiety, and other causes too numerous to mention. We have witnessed it supervene on an attack of schneiderian inflammation, and yield to an eruption of the skin, or on the appearance of a boil. It attacks the trifacial nerve oftenest, because it is the most frequently called into play of any in the body.

When the first branch of the fifth pair of nerves is affected by neuralgia, the complaint is usually denominated *sick headache.* When the facial portions are disturbed, the affection is *tic douleureux.* When the nerves of the stomach are assailed, the disease is *gastralgia* or *gastrodynia.* When the disease is in the nerves of the heart, it is known as *angina pectoris;* elsewhere it is named by the locality, as *sciatica*, *neuralgia mamæ*, *neuralgia pedis.*

The symptoms are very numerous, and would of themselves exhaust the patience to think of them. The principal are throbbing or lancinating pain following the course of the nerve, spasm of the eyes, involuntary weeping, anguish, despondency, confusion of ideas, desire for firm pressure on the head, which will suspend the paroxysms while it is continued.

A neuralgic patient is generally prostrated, and his will enfeebled by his sufferings; desiring others to will, think, and act, without troubling him. A crazy person is often mischievous and malicious, but the neuralgic patient never is.

In the month of March, 1847, the writer was returning from an excursion into Cayuga, Cortland, Chenango, and Madison counties, of this State, when he was attacked with violent pain in the upper jaw on the right side. The cause was periosteal inflammation at the socket of a bicuspid. He had suffered from it before, obtaining relief by a mode of surgical treatment purely original with him. This time he decided upon the removal of the tooth. The pain of the operation was unusually severe and protracted, owing to the inflammation, and the fang of the tooth was of a dark brown tint. Regarding this as the last of his troubles, he neglected taking any special care of himself, and soon became involved in several matters of perplexing business, by which his mind was much distracted and exercised. A relative wishing his aid, he employed himself for several days in work in the woods, sawing logs. The weather was cold, and snow covered the ground. A heavy storm finally set in, and it was not long before he was compelled to desist from his work and take care of himself.

The only medical adviser at hand was my brother, then a student. He declared it a case of influenza, which was then epidemic. I differed from him, and expressed the opinion that it was neuralgia. My conviction was intuitive. It occurred to my mind to say so as he was speaking; but there was not long wanting a heavy pain in the left temple, which steadily increased in intensity, and removed every doubt as to the nature of the malady.

Being partial to water-cure treatment, I wished to employ it; but was over persuaded. My brother gave me a Thompsonian "course of medicine," to which I was repugnant. It gave no relief, but seemed to add to the agony, which had now become excruciating. Lobelia, in "broken doses," afforded some mitigation. The remembrance of that week even now makes me shudder. Every instant as the arteries pulsated, the terrible pain throbbed along the left facial nerve; the ear became deafened, and the left eye, flowing copiously with tears, was presently inflamed as from the sting of a bee. There remained only the consciousness of my own agony. Weary days and sleepless nights were appointed to me.

The roads had been blocked up by the storm, and it had not been practicable to obtain medical advice. A physician, Dr. A. Wood, finally was summoned and managed to break his way to the house. My first appeal to him was inspired by my torture. I entreated him not to burn me up with capsicum, and he obeyed my wish. I apprehend, however, that he found my case a stubborn one; or that I was too much worn down for very acitve treatment. He was a man who would err only on the safer side. He administered an anodyne, which for a short time mitigated the pain. But his other medicines for the next twenty-four hours, failed utterly. The most important ones were not administered promptly as he had directed, and it may have been that there was difficulty in arousing the functions to proper activity.

I remember well one of those fearful nights. I had lost the clear perception of my identity, and imagined myself to be another person, an outcast, in a wilderness, perishing, and at the last extremity. A few faithful ones, I found, were by me, who had forsaken the society of men to share my exile and alleviate my condition. Gratitude for their fidelity was now active in my mind, and regret that I could make them no adequate compensation. But I besought them, as I bade them a last adieu, to take from me whatever valuables I had; for nobody would henceforth care for them as they had cared for me. And all the time that terrible pain darted, every moment, over my temple into the eye and jaw, each throb seeming more fierce than the one that had just preceded. The day finally dawned and I recovered my right mind.

Those six days and nights passed over my head—days and nights of sleepless, unremitting agony. The medicines had accomplished no satisfactory results. The physician redoubled his endeavors. Apocynum was his principal medicine. The throbbing began to lessen in severity. Success was obtained at last, and I slept. Very tedious was my convalescence. For many weeks I was tormented by a stinging sensation along the tract of the affected nerve, which I relieved by binding snow upon the brow and left side of the face. At length a boil appeared on the forehead and when it had discharged its contents the complaint seemed to have passed way. But for many years the sense of hearing in the left ear was greatly benumbed. The track of the nerve was callused, and there was also a blur on the left eye, to keep fresh in my mind the remembrance of that terrible week, and almost to writhe again with its fearful torment.

Subsequent attacks occurred in 1853 and 1856, the former of which was relieved by Dr. H. H. Cator, a homœpathist, with sulphur and kalmia latafolia, and the other by vigorous treatment with opium, chloroform, and brandy.

This complaint occurs most commonly with persons of asthenic condition, or whose powers are broken by advancing years, or who are subject to attacks of rheumatism. Individuals living in a marshy district are liable to it, and, curiously enough, it is there most successfully managed by treating it like fever and ague. Sulphate of quinia, bromide of potassium, and even arsenious acid will control the manifestations. Indeed, medicines of which chlorine, iodine or bromine is an element have been found very successful in neuralgia. Aqua regia or chloro-nitric acid diluted has

3

been regarded my mamy as specific. The old school practitioners have depended on calomel, itself a chloride; although, as Watson shows, and as we have found in other diseases, the sal ammoniac was equally beneficial.

Mr. Abernethy was generally most successful in treating it by directing his attention to the condition of the stomach and bowels. It was his impression that neuralgic patients had a disordered nervous system and morbid condition of the digestive organs. In his peculiar manner, he used to say, "The two are the common parents of a numerous progeny of very dissimilar local diseases. In tic douloureux you must seek to put the digestive organs right, or to soothe the nervous system, according as the one or other may seem to be the principal and primary cause of the disease. Take away one of the parents and there will be no more propagation."

It is far better to use sedatives than to suffer the terrible pain; but there is a more excellent way. Abernethy was right. The disordered condition of the nervous and of the digestive systems occasions neuralgia, catarrh, influenza, and kindred maladies, having different names, are also thus caused; and they all are prone to run into neuralgia. One mode of treatment will cure them all. Relieve the alimentary canal of its contents by apocynum, rhubarb, compound stillingia alterative, or other mild cathartic, adding the bi-carbonate of soda to the medicine; and follow this by a tonic—sulphate of quinia, salicin, some preparation of cohosh, golden seal, or cypripedium, or whatever tonic is acceptable to the patient. Let special pains be taken to get the skin into healthful activity, as this of itself will remedy the entire evil. Much exercise in the sunshine is also important. Neuralgic patients are too much in the shade and in miasmatic, scrofula-breeding atmosphere. The hydrate of chloral is the modern panacea; but it should not be given till carbonate of soda or other antacids have assured a fit condition of the stomach. In acid conditions chloral is often almost inert.

There are scores of remedies as efficient what have been here suggested; and a good physician needs to know them all.—*Am. Eclectic Med. Review.*

